# Gateway reflexes describe novel neuro-immune communications that establish immune cell gateways at specific vessels

**DOI:** 10.1186/s42234-023-00126-1

**Published:** 2023-11-08

**Authors:** Hiroki Tanaka, Rie Hasebe, Kaoru Murakami, Toshiki Sugawara, Takeshi Yamasaki, Masaaki Murakami

**Affiliations:** 1 Division of Molecular Psychoimmunology, Institute for Genetic Medicine and Graduate School of Medicine, Hokkaido University, Kita-15, Nishi-7, Kita-Ku, Sapporo, 060-0815 Japan; 2 Division of Molecular Neuroimmunology, National Institute for Physiological Sciences, national Institute for Natural Sciences, Nishi-38, Myodaiji-cho, Okazaki, 444-8585, Japan; 3 Group of Quantum Immunology, Institute for Quantum Life Science, National Institute for Quantum and Radiological Science and Technology (QST), Anagawa 4-9-1, Inage-Ku, Chiba, 263-8555, Japan; 4 Institute for Vaccine Research and Development (HU-IVReD), Hokkaido University, Nishi-11, Kita-21, Kuta-Ku, Sapporo, 001-0020 Japan

**Keywords:** Neuro-immune communication, Immune regulation, Inflammation, Blood-brain barrier, IL-6 amplifier

## Abstract

Neuroinflammation is an important biological process induced by complex interactions between immune cells and neuronal cells in the central nervous system (CNS). Recent research on the bidirectional communication between neuronal and immunological systems has provided evidence for how immune and inflammatory processes are regulated by nerve activation. One example is the gateway reflex, in which immune cells bypass the blood brain barrier and infiltrate the CNS to cause neuroinflammation. We have found several modes of the gateway reflex in mouse models, in which gateways for immune cells are established at specific blood vessels in the spinal cords and brain in experimental autoimmune encephalomyelitis and systemic lupus erythematosus models, at retinal blood vessels in an experimental autoimmune uveitis model, and the ankle joints in an inflammatory arthritis model. Several environmental stimulations, including physical and psychological stresses, activate neurological pathways that alter immunological responses via the gateway reflex, thus contributing to the development/suppression of autoimmune diseases. In the manuscript, we describe the discovery of the gateway reflex and recent insights on how they regulate disease development. We hypothesize that artificial manipulation of specific neural pathways can establish and/or close the gateways to control the development of autoimmune diseases.

## Introduction

The central nervous system (CNS), which includes the brain and spinal cord, is a unique system with neural connections across several organs and physiological systems (Mai and Paxinos 2011). Bidirectional communication of the CNS with the periphery, such as endocrine systems, digestive organs, gut microbiota, and immune systems, transduce sensory stimuli under psychological disorders into physiological outputs via neural signaling and hormones. In the case of the immune system, chronic psychological stress induces the release of neurotransmitters from axon terminals that can act as paracrine regulators of immune responses including the CNS (Morey et al. 2015).

The CNS is anatomically segregated from other systems and organs by two types of tight barriers: the blood-cerebrospinal fluid (CSF) barrier and the blood-brain barrier (BBB) (Ransohoff and Engelhardt 2012). The blood-CSF barrier forms an apical tight junction between choroid plexus epithelial cells, while the BBB forms a more complicated network of multiple tight junctions between the basal membranes of epithelial cells on brain microvessels (Engelhardt and Sorokin 2009). Immune cells circulate in lymph and blood vessels to perform immunosurveillance against tumor development, bacterial, and viral infection even in the CNS, although their access to the CNS is significantly limited by the BBB. Cells including microglia within the CNS parenchyma exhibit low MHC class II expression, indicating that the CNS establishes a specialized environment in which T cells are difficult to activate (Bö et al. 1994). T cells are one of the main causes of tissue inflammation and autoimmunity and exist in the CSF (Kivisakk et al. 2003). The presence of the BBB suggests that the CNS is an immune-privileged site and that the adaptive immune system is severely restricted there.

However, recent studies have challenged this idea about the immune privilege of the CNS. The CNS is a common target of viral infections and autoimmune neuroinflammatory diseases (Korn and Kallies 2017). The self-antigens released from damaged parenchymal CNS tissues are drained into lymphoid organs through the CSF, leading to neurodegeneration and neuroinflammation (Locatelli et al. 2012). Furthermore, immune cells encounter many blood vessels in the CNS after they recognize parenchyma-derived antigens drained from the CNS (Louveau et al. 2015). These findings highlight the importance of immune cells in the CNS during immunosurveillance as well as the development of neuroinflammation. The gateway reflex is a new concept of neuro-immune interactions, in which immune cells, including autoreactive CD4+ T cells, respond to CNS antigens in the blood via formation of ‘the gateway’ for immune cell in blood vessels (Kamimura et al. 2020; Uchida et al. 2022). Additionally, recent studies have revealed that the gateway formation leads to different types of biological response from autoimmunity. In this review, we describe the structural and functional feature of the gateway reflex and how it connects specific neural activation depending on external or internal stimuli to modulate the immune responses.

## T cells and neural circuits in the CNS

Experimental autoimmune encephalomyelitis (EAE) is a widely used model for multiple sclerosis (MS), which is a neuroinflammatory disease characterized by focal lymphatic infiltration and demyelination in the CNS followed by the development of neurological and psychiatric disorders (Constantinescu et al. 2011). In EAE, the immunization of CNS antigens with their peptide fragments or whole proteins, such as myelin-derived proteins, induces the expansion of autoimmune myelin-specific CD4+ T cells followed by neuroinflammation development. The autoreactive CD4+ T cells highly express chemokine receptors and cell adhesion molecules to interact with chemokines and ICAM1 on the endothelial cells of the gateway of immune cells and to infiltrate infiltrate from blood vessels into the CNS (Yednock et al. 1992). T cell migration across the BBB is followed by infiltration in the CNS and the development of neuroinflammation. Neuroinflammation in the CNS leads to the dysregulation of neuronal circuits around myelinated spinal axons and progressive clinical signs such as tail and hindlimb paralysis (Constantinescu et al. 2011). EAE pathology appears not only by the immunization of myelin-derived peptides (active EAE model), but also by the intravenous transfer of activated myelin-specific CD4+ T cells, which are re-stimulated in vitro with myelin oligodendrocyte glycoprotein (MOG) peptide, into wild-type C57BL/6 mice (adoptive transfer EAE (tEAE) model) (Stromnes and Goverman 2006). In tEAE, T_H_17 cells, a subset of activated CD4+ T cells, are required for EAE progression (Langrish et al. 2005). Myelin-specific T_H_17 cells from immunized mice or transgenic mice with MOG peptide-specific TCR can be maintained and expanded in vitro by TCR signaling via antigen presentation in the presence of interleukin (IL)-23, which is required for the survival of T_H_17 cells (Stromnes and Goverman 2006; Jager et al. 2009). Activated T_H_17 cells highly secrete proinflammatory cytokines, including IL-17, TNF-α and IL-6, which contribute to the inflammatory response of nonhematopoietic cells by synergistically amplifying inflammation in combination with the simultaneous activation of STAT3 and NFkB (Ogura et al. 2008; Shen et al. 2006). A mechanistic scheme involving T_H_17 cell-associated inflammation around the BBB is a widely accepted hypothesis for how autoreactive T cells by this boundary cause autoimmune diseases (Hirota et al. 2007; Liston et al. 2009).

Numerous studies have demonstrated the pivotal role of T_H_17 cells and IL-17 in the pathogenesis and development of chronic autoimmune diseases, and clinical strategies have accordingly been adopted to target T_H_17 cells and IL-17 (Miossec and Kolls 2012; Gaffen et al. 2014). In chronic inflammation, IL-17 enhances the innate immunity of nonhematopoietic cells, such as synovial fibroblasts for rheumatoid arthritis, keratinocytes for psoriasis, and vascular endothelial cells in EAE (Kim et al. 2007; Arima et al. 2012; Furue et al. 2020). Although IL-17 is a relatively weak activator of the NK-κB pathway, its cooperative stimulation with IL-6 leads to the overproduction of cytokines and chemokines in a feedback loop of NK-κB and STAT3, a phenomenon termed the IL-6 amplifier (Ogura et al. 2008; Lee et al. 2012). Cytokines, chemokines, and growth factors in this inflammatory cycle promote the accumulation of immune cells, including pathogenic T_H_17 cells, into local inflammatory lesions, accelerating local damage to peripheral organs. Thus, the IL-17-mediated positive feedback of inflammation provides insights into the mechanism underlying the exacerbation of chronic diseases associated with T_H_17 cells. Along with IL-17, activated CD4+ T cells express IL-6 and TNFa, two NF-kB activators that enhance the IL-6 amplifier (Ogura et al. 2008; Lee et al. 2012). The IL-6 amplifier induces a massive release of several types of chemokines and growth factors from nonhematopoietic cells in Rheumatoid arthritis and EAE model (Harada et al. 2015), able to attract inflammation-associated immune cells such as T cells, neutrophils, inflammatory monocytes, and macrophages and to promote overgrowth of fibroblasts in affected tissues. These severe inflammation causes ankylosis in the large joints (Atsumi et al. 2002). So the prolonged and amplified inflammation of nonhematopoietic cells by the IL-6 amplifier will become fundamental concept to understand the complex mechanism of tissue destruction during disease onset and development of autoinflammatory diseases.

Nervous systems orchestrate most physiological processes to maintain cell and tissue homeostasis. The disturbance of neural circuits can cause the dysfunction of multiple systems including the immune system. For example, the hypothalamic-pituitary-adrenal (HPA) axis releases glucocorticoids systemically upon psychological stresses; these hormones suppress immune cell functions (Bellavance and Rivest 2014). Another example is how the stress mediator corticotropin-releasing hormone (CRH) induces mast cell degranulation and enhances vascular permeability in skin tissue (Theoharides et al. 1998). CRH also activates skin fibroblasts and keratinocytes to release several inflammatory mediators that selectively suppress T_H_1 responses and promote a skewing toward allergic T_H_2 responses (Arck et al. 2006).

The vagus nerve pathway mainly provides parasympathetic innervation to digestive and endocrine organs. Notably, this pathway induces the anti-inflammatory vagal reflex by releasing neurotransmitters from the vagus nerve to immune cells (Breit et al. 2018; Bonaz et al. 2016). Additionally, neural activation under physiological stress indirectly affects vascular inflammation through T cell activation. Finally, psychological stresses activate the renin-angiotensin-aldosterone cascade to release angiotensin II, a strong activator for T cells, which produce the several cytokines required for vascular inflammation and immune cell recruitment (Dinh et al. 2014; Guzik et al. 2007). Previous studies demonstrated an association of the sensory nerve pathway with regulation of inflammatory processes via an antidromic way. Indeed, antidromic stimulation of capsaicin-sensitive vagal and splanchnic afferent (C- and Aδ-fibers) pathways results in the release of neuropeptides such as calcitonin gene-related peptide (CGRP) from sensory nerve axon terminal (Holzer 1988). These substances have a dual role in inflammation, they contribute to protective reflexes in the gastrointestinal and nasal mucosa (Holzer 1991), while the innervation via efferent and afferent/sensory fibers rather than vagus nerves is important for protection of gut in DSS-induced mouse colitis model (Willemze et al. 2018).

## Gateway reflex

The gateway reflex is a group of neural and immune responses that create gateways at specific blood vessels for immune cells to infiltrate nervous systems. They regulate local neuroinflammation and affect neuro-immune functions as well as some clinical aspects of neuroimmune diseases.

### Gravity gateway reflex

Weight-bearing muscles, joints, and bones are affected by gravity. Soleus muscles, which are anti-gravity muscles and necessary to maintain posture for weight-bearing, continuously stimulate neural circuits in both humans and mice. Dorsal root ganglia (DRG) at the fifth lumbar spinal cord (L5) spinal cord are innervated by sensory neurons of the soleus muscles. Gravity stimulation can constantly activate the neighboring spinal cords of dorsal vessels through the innervation of sensory neurons from the L5 DRG to create the gravity gateway reflex via the sympathetic pathway from L5 sympathetic ganglions.

**Fig. 1:**
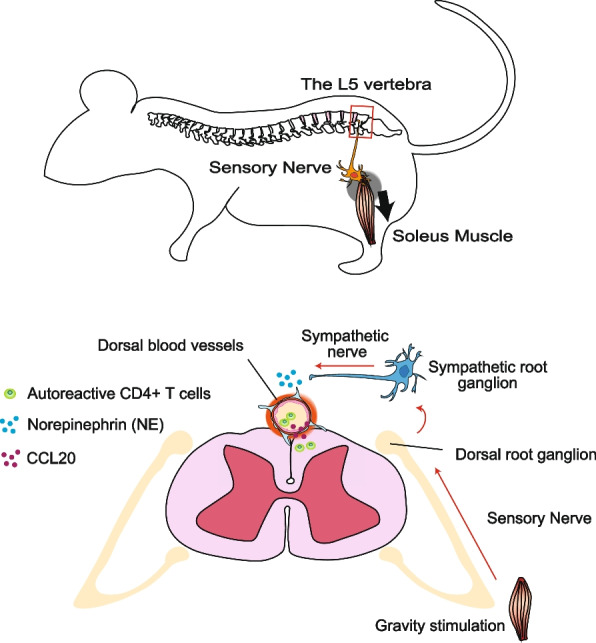
Gravity gateway reflex. Gravity activates sensory nerves in the soleus muscle, whose cell bodies are located at the dorsal root ganglion of the fifth lumbar (L5) spinal cord (upper). The retrograde activation of sensory nerves propagates into sympathetic ganglions via an unknown pathway to release norepinephrine (NE) from the peripheral axons of sympathetic nerves to L5 dorsal blood vessels. NE amplifies inflammation initiated by autoreactive (MOG-specific) T cells at the L5 blood vessels in an IL-6 amplifier-dependent manner. It also enhances the release of CCL-20 and infiltration of autoreactive T cells inside the CNS (bottom)

To explore how autoreactive CD4+ T cells against myelin-peptides accumulate in the CNS, we employed the tEAE model, a mouse model for MS induced by the intravenous injection of MOG-primed CD4+ T cells (Langrish et al. 2005; Ogura et al. 2008). The disease in tEAE develops in a similar fashion as that in the active EAE model, including the loss of tail tonicity and development of hindlimb paralysis, despite tEAE being free of complete Freund’s adjuvant and pertussis toxin. Given the widespread distribution of myelin in the CNS and perivascular circulation of myelin-specific autoreactive CD4+ T cells, there exists a unique entrance site to the CNS for these T cells. We found that myelin-specific CD4+ T cells preferably accumulate in the dorsal vessels of the L5 cord at the onset of EAE symptoms (Arima et al. 2012) (Fig. 1). The accumulation of these CD4+ T cells induces strong inflammation around the L5 dorsal vessels to drive the IL-6 amplifier. CCL20, a chemokine that attracts CCR6-expressing immune cells, such as T_H_17 cells (Hirota et al. 2007; Borgne et al. 2006), is released from the inflamed dorsal vascular endothelial cells, promoting the assembly of myelin-specific CD4+ T cells at L5 dorsal vessels.

Suspending mice by their tails unloads the hindlimbs to reduce gravity stimulation to the soleus muscles. Tail suspension significantly reduced T cell numbers and CCL20 production in L5 dorsal endothelial cells during tEAE onset (Arima et al. 2012). Tail suspension also inhibited the expression of c-fos, an indicator of neural activation, in L5 DRG (Arima et al. 2012). This immunosuppressive effect is canceled by the electrical stimulation of the soleus muscles, resulting in CCL20 expression from the L5 dorsal vessels of tEAE mice even with tail suspension (Arima et al. 2012). Gravity stimulation released norepinephrine (NE), a neurotransmitter and NFkB stimulator, from sympathetic neurons around the L5 dorsal vessels to induce inflammatory cytokines via the IL-6 amplifier (Arima et al. 2012) and enhance inflammation by increasing the release of CCL20 in endothelial cells of the L5 dorsal vessels (Fig. 1). Thus, the gravity gateway reflex is a mechanism by which sensory-sympathetic crosstalk controls regional blood vessels to produce cytokines and chemokines, leading to the accumulation of autoreactive T cells in the blood, their entry into the CNS, and the development of an MS-like pathogenesis. As well as myelin-specific CD4+ T cells, myelin-specific CD8+ cytotoxic T cells can infiltrate into the CNS and induce brain neuroinflammation, although the major sites for their entry are located at brain but not at the spinal cord (Huseby et al. 2001). This indicates the different mechanism of CD8+ T cell entry into the CNS.

### Pain-induced gateway reflex

**Fig. 2:**
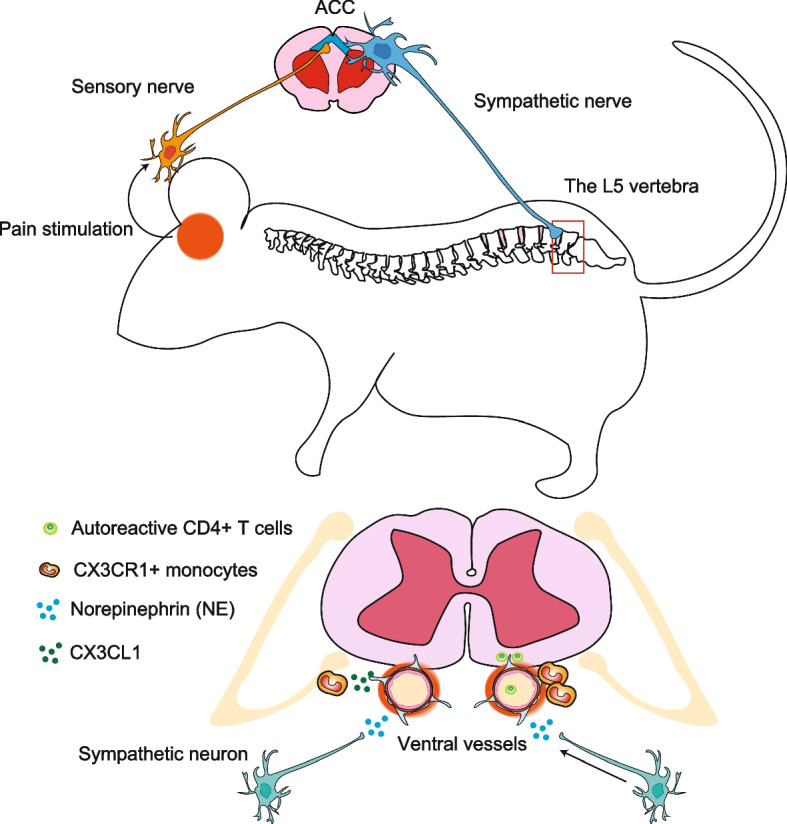
Pain-induced gateway reflex. Upon sensing pain, sensory nerves propagate activation signals to the anterior cingulate cortex (ACC), a key region for pain processing in the brain. This activation finally reaches the L5 vertebrate via sympathetic nerves to induce CX3CL1 release from ventral blood vessels in a norepinephrine-dependent manner. CX3CL1 recruits MHC class II^hi^ monocytes around the ventral vessels. In EAE mice experiencing remission 20 days after the intravenous injection of MOG specific CD4+ T cells, pain stimulation induces the re-activation of MOG-specific CD4+ T cells at the ventral vessels and their infiltration into the CNS followed by the relapse of EAE disease symptoms with CNS neuroinflammation

Pain is an unpleasant sensory and emotional experience associated with actual and potential tissue damage (Raja et al. 2020). Upon a peripheral injury, immune cells can activate nociceptors on neuronal axons to express inflammatory mediators that trigger pain signals and tissue inflammation (Ren and Dubner 2010). We found that pain induction by the partial ligation of the middle part of the trigeminal nerve induces the recurrence of EAE during the remission phase in tEAE (Arima et al. 2015) (Fig. 2). Pain stimuli are delivered to the anterior cingulate cortex (ACC), which has neurons related to pain sensation, and subsequently activate sympathetic circuits distributed to ventral vessels of the spinal cord, creating the pain-induced gateway at specific sites around the L5 spinal cord during the remission phase of EAE (Arima et al. 2015). After the pain sensation, NE is released from sympathetic axons around whole ventral vessels of the spinal cord to activate the IL-6 amplifier. Because there are MHC class II+ monocytes at the L5 sites that accumulated from the blood during the initial disease development, CX3CL1 secretion from the vessels and MHC class II+ monocytes themselves accumulate MHC class II+ monocytes around the L5 ventral vessels to increase vessel permeability. Circulating MOG-specific autoreactive CD4+ T cells in the blood also accumulate at the L5 ventral vessels to develop EAE relapse (Arima et al. 2015) (Fig. 2). The numbers of inflammatory monocytes accumulated to peripheral of the meninges of the spinal cords have a correlation of the disease severity of EAE (Ajami et al. 2011), supporting our findings with MHC class II+ monocytes accumulation required for EAE relapse. Thus, pain stimuli during the remission phase of tEAE established immune cell-gateways in the presence of MHC class II+ monocytes in the L5 cord. Notably, MS patients show a very characteristic pattern of periodic relapse followed by remission (Steinman 2014). Therefore, the proposed mechanism of the pain-gateway reflex may explain the clinical pattern of relapse and remission in MS patients.

### Stress gateway reflex

**Fig. 3:**
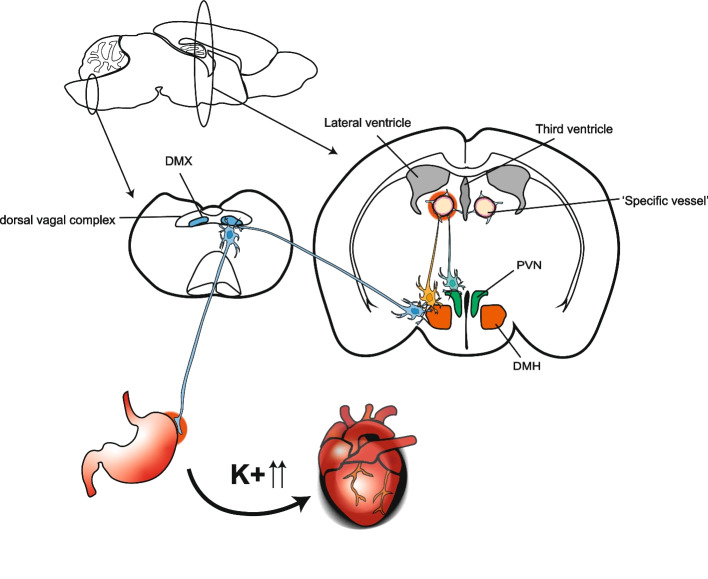
Stress-induced gateway reflex. Chronic mental stresses sequentially activate neurons in the paraventricular nucleus of the hypothalamus (PVN) and tyrosine hydroxylase neurons connecting specific vessels adjacent to the third ventricle, dentate gyrus, and thalamus. This neural activation induces the CCL5-expression-dependent accumulation of CD4+ T cells and MHC class II^hi^ monocytes followed by microinflammation in specific brain vessels. The microinflammation activates the dorsomedial nucleus of hypothalamus (DMH) via neural connections, propagates activation signals to dorsal motor nucleus of the vagus nerve (DMX), and finally causes upper severe gastrointestinal (GI) tract failure. Bleeding from the upper GI tract elevates blood potassium ion levels, which are associated with myocyte apoptosis and an increased mortality in acute heart failure

Chronic stresses are known to be a significant risk factor for gastrointestinal, cardiovascular, and autoimmune diseases. Anxiety, depression, and posttraumatic stress disorder progressively increase a risk of inflammatory bowel diseases in a national cohort of US veterans (Thakur et al. 2020). Chronic stresses include those that activate neural pathways toward the paraventricular nucleus (PVN), dorsomedial hypothalamus (DMH), dorsal motor nucleus of the vagus verve (DMX), and vagus nerves (Ulrich-Lai and Herman 2009). Efferent activation of the parasympathetic and vagal nerves under psychological stresses develops the gut-associated inflammation in acute and chronic gastroenteritis (Browning and Travagli 2014; Kenney and Ganta 2014). Accordingly, we investigated the chronic stress-mediated gateway reflex in the tEAE model. For this experiment, we employed sleep disturbance stress (SDS) and stress induced by an unfavorable circumstance, where an individual mouse was housed in the special cage for providing perpetual avoidance of water on a wheel (PAWW) stress (Miyazaki et al. 2013). tEAE mice under these chronic stresses did not exhibit any typical symptoms of the EAE pathology, such as hindlimb paralysis, but they did show acute upper gastrointestinal failure with the removal of epidermal and mucus layers and bloody stool. Additionally, they exhibited an increase in blood potassium ion concentration, leading to fatal cardiovascular damage with sudden death (Arima et al. 2017) (Fig. 3). Because there was almost no autoreactive CD4+ T cells in the L5 cord, we concluded that chronic stress opened a different immune cell gateway from the one caused by gravity. In the stressed tEAE model, autoreactive CD4+ T cells had accumulated at blood vessels adjacent to the dentate gyrus, thalamus, and third ventricle. Additionally, dopaminergic (tyrosine hydroxylase positive; TH+) neurons distributed around specific blood vessels were activated in the PVN, a direct stress response site (Arima et al. 2017). The activated neurons secreted NE to stimulate the IL-6 amplifier at the specific vessels to recruit autoreactive CD4+ T cells from blood together with MHC class IIhi monocytes followed by the amplification of local inflammation. Microinflammation around the blood vessels released extracellular ATP, which acts as a neurotransmitter for DMX neurons around specific vessels through ATP receptors (Fig. 3). In the stress gateway reflex, microinflammation at specific vessels in the brain and the ATP-induced neural activation stimulated the vagus nerve pathway, which caused epithelial damage in the stomach through the acetylcholine-dependent overproduction of gastric acid. The epithelial cell damage induced bleeding at the upper gastrointestinal tract and the acute elevation of cytosolic potassium ions, followed by sudden cardiac dysfunction and death. PVN and DMH are also key regulators of circulatory control by regulating blood pressure (Guyenet 2006). Activation of sympathetic nervous system under chronic stresses will assist elevated blood pressure and contribute to the stress-induced mortality by promoting the bleeding at GI tract. Thus, chronic stress is a key factor in the bidirectional communication between the nervous and immune systems. Brain neuroinflammation is a strong risk factor for the development of neurodegenerative disease including Alzheimer’s disease, non-Alzheimer’s dementia, Parkinson’s diseases, and epilepsy (Chen et al. 2016; Lull and Block 2010). We have also demonstrated that inflammation at specific blood vessels of the brain during the stress gateway reflex is an important prognostic factor for acute gastrointestinal diseases and cardiovascular death (Arima et al. 2017).

### Light gateway reflex

**Fig. 4:**
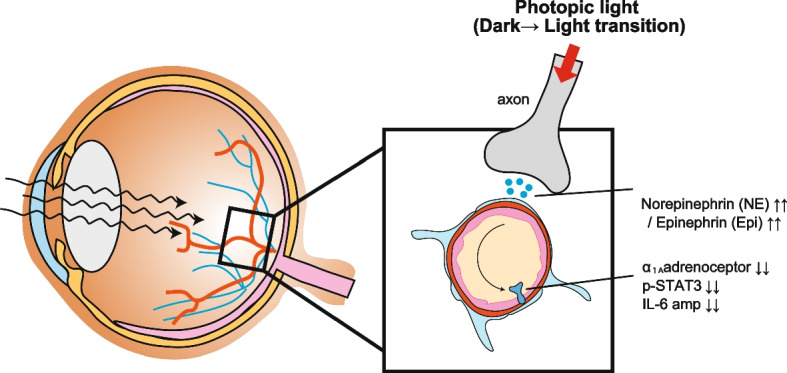
Light gateway reflex. The retinal inflammation found in experimental autoimmune uveitis (EAU) model mice is strongly attenuated by photopic light illumination. Photopic light stimulation induces the excessive release of norepinephrine (NE) and epinephrine (Epi) from retinal neurons to retinal blood vessels, resulting in the downregulation of α_1A_ adrenergic receptors. In retinal blood vessels, NF-κB and STAT-3 activation is inhibited, and IL-6 production is reduced. This suppressive response contributes to a reduced number of immune cells infiltrating the retina, thus attenuating EAU

Uveitis is an autoimmune disease with inflammation and tissue damage mediated by autoreactive CD4+ T cells in the retina and choroid and can lead to significant visual deficiency and blindness if untreated (Caspi 2010). Experimental autoimmune uveitis (EAU) is a mouse model for human uveitis (Caspi 2010; Caspi 2003). EAU is induced by immunization with a peptide antigen derived from human photoreceptor retinoid binding protein (hIRBP) with complete Freund’s adjuvant and pertussis toxin. hIRBP-specific (uveitogenic) CD4+ T cells infiltrate the retinal tissue across the blood-retina barrier, which is the retina’s blood barrier, to cause progressive inflammation in the retina (Stofkova et al. 2019). In EAU, activated uveitogenic T cells accumulate in retinal blood vessels 10 days after immunization, where immune cells form gateways in the retina (Stofkova et al. 2019). However, mice with EAU pathology showed fewer T cells in the retina after exposure to photopic light, which stimulates strongly neurons in retina tissues (Stofkova et al. 2019). Photopic light during EAU inhibits adrenergic receptor expression on endothelial cells of retinal blood vessels, making these cells less sensitive to NE, thus reducing inflammation and cytokine production and failing to activate the IL-6 amplifier. It is known that bright light has protective effects on retinal nerves and photoreceptors by producing neuroprotective cytokines and antioxidants from damaged cells (Fig. 4) (Organisciak and Vaughan 2010). Thus, our model revealed the role of photopic illumination in protecting retinal organs from autoreactive T cell infiltration to prevent neuroinflammation and tissue damage. The light gateway reflex is a novel gateway reflex, because it negatively regulates injured vascular endothelial cells to provide a protective role.

### Remote inflammation gateway reflex

**Fig. 5:**
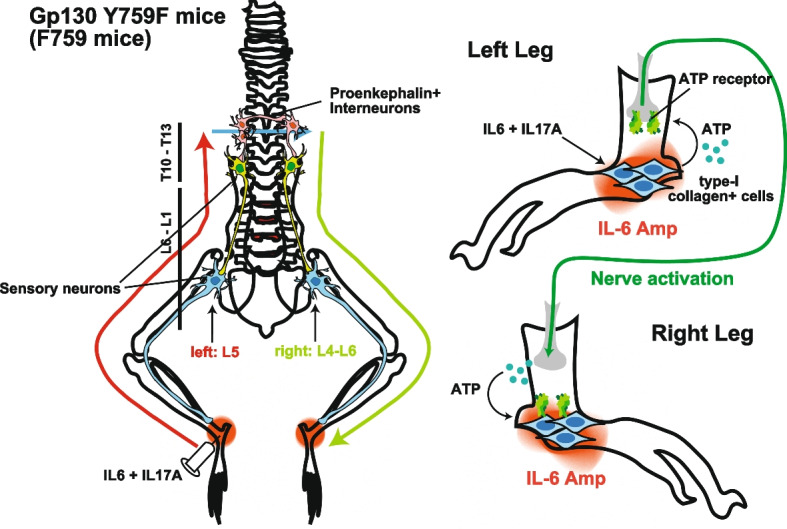
Remote inflammation gateway reflex. A schematic model of the remote gateway reflex in the symmetrical spread of inflammatory lesions. The stimulation of collagen type I+ ankle nonhematopoietic cells from gp130 Y759F mutant (F759) mice with proinflammatory cytokines IL-6 and IL-17 strongly activates NF-κB and STAT-3 in an IL-6 amplifier-dependent manner, releasing extracellular ATP as a neurotransmitter. Sensory activation primed by sensitization to the ATP receptor P2X7 propagates from one side of the DMX to the other side through nerve networks comprised of sensory neurons and proenkephalin+ interneurons. Sensory stimulations arriving at the contralateral terminal promote ATP release from the sensory nerves to drive NF-κB and STAT-3 activation-mediated inflammation in collagen type I+ nonhematopoietic cells located at the contralateral side of the original inflammatory lesions

Inflammatory arthritis, including rheumatoid arthritis and psoriatic arthritis, are characterized by joint inflammation, joint swelling, pain at the joints, and destruction of synovial joints (Aletaha et al. 2010). In a certain group of inflammatory arthritis patients, joint inflammation occurs bilaterally along the affected joints (Aletaha et al. 2010; Guo et al. 2018). Consistently, several animal models of rheumatoid arthritis often show bilateral spreading inflammation within joints (Atsumi et al. 2002; Sakaguchi et al. 2003; Tuncel et al. 2016). Some studies have suggested the important role of neural pathways in the bilateral spread of inflammation during arthritis development (Donaldson et al. 1995; Kidd et al. 1989). We revealed that neural pathways connecting both ankle joints are critical for the inflammation spreading in a symmetrical manner. We defined these neural pathways as the remote inflammation gateway reflex (Hasebe et al. 2022). gp130^F759/F759^ (F759) mice are a mouse model that spontaneously develops rheumatoid arthritis in which joint inflammation is amplified by the IL-6 amplifier (Ogura et al. 2008; Atsumi et al. 2002). In F759 mice, arthritis can be rapidly induced by administering IL-6 and IL-17A to the joints (Harada et al. 2015; Murakami et al. 2011). In the remote inflammation gateway reflex, the bilateral spread of inflammation is accomplished by neural crosstalk between sensory and interneurons that utilize ATP both as a neurotransmitter and as an inflammation enhancer. To test how inflammation-induced neural activation is transmitted through neural circuits and expanded to the peripheral sites, we employed two arthritis models, F759 mice and type II collagen-immunized mice, with priming arthritic inflammation and neural activation between both ankle joints (Hasebe et al. 2022). ATP released from non-immune cells during inflammation stimulated sensory pathways. The activation signal in neurons propagated primarily through neural circuits composed of Nav1.8+ sensory neurons and proenkephalin+ interneurons to reach the contralateral ankle joints, in which ATP was released from the sensory axon and promoted inflammation via the IL-6 amplifier (Fig. 5). An ATP receptor antagonist (A438079) or surgical dissection of Nav1.8+ sensory and proenkephalin+ interneuron networks successively inhibited the symmetrical spread of inflammation in these models (Hasebe et al. 2022). These results have only been demonstrated in mice but encourage further studies to explore therapeutic targets to treat diseases with symmetrical inflammation spreading such as rheumatoid arthritis.

### Immunological and behavioral modification of SLE model mice under stress

**Fig. 6:**
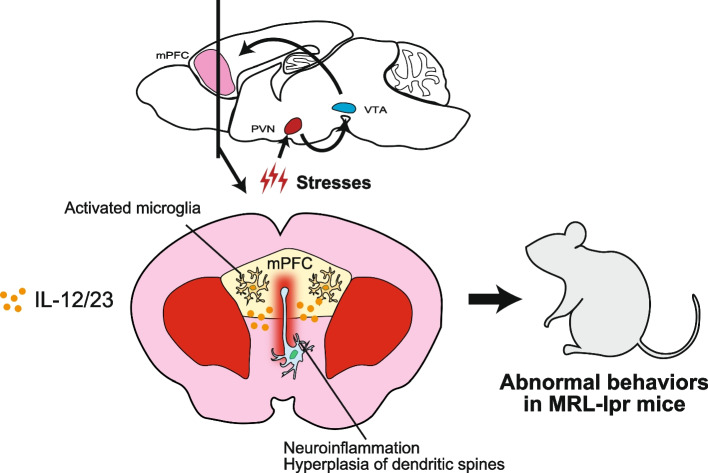
Sleep disturbance stress (SDS)-subjected MRL/lpr mice as a model of neuropsychiatric SLE (NPSLE). Under SDS, MRL/lpr mice show disinhibited anxiolytic behaviors. SDS induces sequential nerve activation in the paraventricular nucleus of the hypothalamus (PVN), the ventral tegmental area (VTA) and medial prefrontal cortex (mPFC) to release IL-12/23 p40 from microglia in the mPFC. This cytokine excessively activates brain neurons to induce the hyperplasia of dendritic spines in the mPFC. SDS-subjected MRL/lpr mice demonstrate patterns of disease symptoms in NPSLE patients including high IL12/23 p40 production in the cerebrospinal fluid (CSF) and atrophy in the mPFC. Anti-IL12/23 p40 challenges improve the clinical features between SDS-subjected MRL/lpr mice and human NPSLE, indicating the pathological roles of IL12/23 p40 in these psychological disorders

Systemic lupus erythematosus (SLE) is an autoimmune disease attributed to the loss of immunological tolerance with many symptoms including skin rash, arthritis, nephritis, hematologic abnormalities, and inflammation in the CNS (Tsokos 2011). About half of SLE patients show some kind of neurological and psychiatric symptoms, including headaches, mood disorder, anxiety, and mild cognitive disfunctions, which are diagnosed as neuropsychological SLE (NPSLE) (Schwartz et al. 2019). NPSLE is further classified into focal neurological syndrome-type NPSLE (fNPSLE) and diffuse neurological-psychiatric-cognitive syndrome-type NPSLE (dNPSLE) (Ellis and Verity 1979). dNPSLE is diagnosed in 80% of NSLE patients and is characterized by diverse clinical manifestations including cerebrovascular disease, seizure disorder, acute confusional state (ACS), and neuropathies. It is hypothesized that most cases have dysregulated neural circuits caused by autoimmunity and autoinflammatory conditions in the CNS, but there is still no NPSLE mouse model to study the disease. On the other hand, several mouse models exist for the disease manifestation and pathogenesis of human SLE. MRL/MpJ^*lpr/lpr*^ (MRL-lpr) mice spontaneously develop autoimmune cell infiltration and systemic inflammation in multiple organs (Theofilopoulos and Dixon 1985; Vogelweid et al. 1991). We hypothesized that chronic stress, such as SDS, in this SLE model causes NPSLE-like behaviors. Notably, MRL-lpr mice under SDS display risk-taking agitation-like behavior, less anxiety-like behavior, hyperactivity, and psychomotor agitation compared with mice without stress (Abe et al. 2022). Further, SDS activates microglia in the medial prefrontal cortex (mPFC), an important coordinator of behavioral and psychological stress in the forebrain (McKlveen et al. 2015). Additionally, these mice express high levels of the inflammatory cytokine gene IL-12/IL23 p40 (Fig. 6). IL12/23 from microglia activates neurons in the mPFC to form an abnormal number of dendritic spines that may lead to the hyperactivation of neurons, potentially causing the dysregulation of neural circuits in the CNS (Abe et al. 2022; Kaufmann and Moser 1991). Neurological pathologies in the mPFC may explain why SDS-subjected MRL-lpr mice show disinhibited behaviors like agitation, psychosis, and ACS. Interestingly, the enhanced expression of IL-12/IL23 p40 in the CSF and atrophy in the mPFC are prominent in patients with dNPSLE, suggesting neural and microglial hyperactivation like that observed in SDS-subjected MRL-lpr mice (Abe et al. 2022). Further, the administration of IL-12R (a common receptor for IL-12 and IL-23)-blocking antibodies and tyrosine kinase-2 inhibitors reduced abnormal behaviors and dendritic spine hyperplasia in SDS-subjected MRL/lpr mice (Abe et al. 2022). Therefore, our observations indicate a positive correlation between dNPSLE and the SDS-subjected MRL-lpr model in an immunological and clinical point of view. Following these findings, IL-12/IL23 p40 mainly released from the mPFC is a potential therapeutic target for dNPSLE.

## Conclusion

The gateway reflex describes neuro-immune interactions at specific boundaries between the CNS and adjacent blood vessels during inflammatory and immune events. Several types of environmental stimulations, including gravity, pain, psychological stress, and light, activate specific neural pathways to establish immune cell gateways at specific blood vessels including the CNS and cause tissue-specific inflammatory diseases. From a clinical point of view, the manipulation and control of the gateway reflex are potential management strategies for some inflammatory diseases. Recent advances in techniques for the artificial control of neural activation, such as chemogenetics and optogenetics, may be used to activate specific neuronal projections leading to neurological and psychological manifestations (Roth 2016; Bernstein and Boyden 2011), raising the possibility of manipulating brain microinflammation during the gateway reflex. Blockade for neuropeptides or extracellular ATP binding to their receptors in the stress gateway reflex will become novel therapeutic strategies to prevent stress-induced mortality or NPSLE. We expect that novel strategies to artificially modulate the gateway reflex will provide treatments for chronic inflammatory diseases and cancers by inhibiting or accelerating immune cell entry into the CNS.

## Data Availability

Not appreciable.

## References

[CR1] Abe N, Tarumi M, Fujieda Y, Takahashi N, Karino K, Uchida M, Kono M, Tanaka Y, Hasebe R, Kato M (2022). Pathogenic neuropsychiatric effect of stress-induced microglial interleukin 12/23 axis in systemic lupus erythematosus. Ann Rheumatic Dis.

[CR2] Ajami B, Bennett JL, Krieger C, McNagny KM, Rossi FM (2011). Infiltrating monocytes trigger EAE progression, but do not contribute to the resident microglia pool. Nat Neurosci.

[CR3] Aletaha D, Neogi T, Silman AJ, Funovits J, Felson DT, Bingham CO, Birnbaum NS, Burmester GR, Bykerk VP, Cohen MD (2010). 2010 Rheumatoid arthritis classification criteria: an American College of Rheumatology/European League Against Rheumatism collaborative initiative. Arthritis Rheum.

[CR4] Arck PC, Slominski A, Theoharides TC, Peters EM, Paus R (2006). Neuroimmunology of stress: skin takes center stage. J Invest Dermatol.

[CR5] Arima Y, Harada M, Kamimura D, Park JH, Kawano F, Yull FE, Kawamoto T, Iwakura Y, Betz UA, Marquez G (2012). Regional neural activation defines a gateway for autoreactive T cells to cross the blood-brain barrier. Cell.

[CR6] Arima Y, Kamimura D, Atsumi T, Harada M, Kawamoto T, Nishikawa N, Stofkova A, Ohki T, Higuchi K, Morimoto Y, et al. A pain-mediated neural signal induces relapse in murine autoimmune encephalomyelitis, a multiple sclerosis model. Elife. 2015;4:e08733. 10.7554/eLife.08733. 10.7554/eLife.08733 PMC4530187 26193120

[CR7] Arima Y, Ohki T, Nishikawa N, Higuchi K, Ota M, Tanaka Y, Nio-Kobayashi J, Elfeky M, Sakai R, Mori Y, et al. Brain micro-inflammation at specific vessels dysregulates organ-homeostasis via the activation of a new neural circuit. Elife. 2017;6:e25517. 10.7554/eLife.25517. 10.7554/eLife.25517 PMC5557598 28809157

[CR8] Atsumi T, Ishihara K, Kamimura D, Ikushima H, Ohtani T, Hirota S, Kobayashi H, Park SJ, Saeki Y, Kitamura Y (2002). A point mutation of Tyr-759 in interleukin 6 family cytokine receptor subunit gp130 causes autoimmune arthritis. J Exp Med.

[CR9] Bellavance MA, Rivest S (2014). The HPA - Immune Axis and the Immunomodulatory Actions of Glucocorticoids in the Brain. Front Immunol.

[CR10] Bernstein JG, Boyden ES (2011). Optogenetic tools for analyzing the neural circuits of behavior. Trends Cogn Sci.

[CR11] Bö L, Mörk S, Kong PA, Nyland H, Pardo CA, Trapp BD (1994). Detection of MHC class II-antigens on macrophages and microglia, but not on astrocytes and endothelia in active multiple sclerosis lesions. J Neuroimmunol.

[CR12] Bonaz B, Sinniger V, Pellissier S (2016). Anti-inflammatory properties of the vagus nerve: potential therapeutic implications of vagus nerve stimulation. J Physiol.

[CR13] Breit S, Kupferberg A, Rogler G, Hasler G (2018). Vagus Nerve as Modulator of the Brain-Gut Axis in Psychiatric and Inflammatory Disorders. Front Psychiatry.

[CR14] Browning KN, Travagli RA (2014). Central nervous system control of gastrointestinal motility and secretion and modulation of gastrointestinal functions. Compr Physiol.

[CR15] Caspi RR (2003). Experimental autoimmune uveoretinitis in the rat and mouse. Curr Protoc Immunol.

[CR16] Caspi RR (2010). A look at autoimmunity and inflammation in the eye. J Clin Invest.

[CR17] Chen WW, Zhang X, Huang WJ (2016). Role of neuroinflammation in neurodegenerative diseases (Review). Mol Med Rep.

[CR18] Constantinescu CS, Farooqi N, O'Brien K, Gran B (2011). Experimental autoimmune encephalomyelitis (EAE) as a model for multiple sclerosis (MS). Br J Pharmacol.

[CR19] Dinh QN, Drummond GR, Sobey CG, Chrissobolis S (2014). Roles of inflammation, oxidative stress, and vascular dysfunction in hypertension. BioMed Res Int.

[CR20] Donaldson LF, McQueen DS, Seckl JR (1995). Neuropeptide gene expression and capsaicin-sensitive primary afferents: maintenance and spread of adjuvant arthritis in the rat. J Physiol.

[CR21] Ellis SG, Verity MA (1979). Central nervous system involvement in systemic lupus erythematosus: a review of neuropathologic findings in 57 cases, 1955–1977. Semin Arthritis Rheum.

[CR22] Engelhardt B, Sorokin L (2009). The blood-brain and the blood-cerebrospinal fluid barriers: function and dysfunction. Semin Immunopathol.

[CR23] Furue M, Furue K, Tsuji G, Nakahara T. Interleukin-17A and Keratinocytes in Psoriasis. Int J Mol Sci. 2020;21(4):1275. Available at 10.3390/ijms21041275. 10.3390/ijms21041275 PMC7072868 32070069

[CR24] Gaffen SL, Jain R, Garg AV, Cua DJ (2014). The IL-23-IL-17 immune axis: from mechanisms to therapeutic testing. Nat Rev Immunol.

[CR25] Guo Q, Wang Y, Xu D, Nossent J, Pavlos NJ, Xu J (2018). Rheumatoid arthritis: pathological mechanisms and modern pharmacologic therapies. Bone Res.

[CR26] Guyenet PG (2006). The sympathetic control of blood pressure. Nat Rev Neurosci.

[CR27] Guzik TJ, Hoch NE, Brown KA, McCann LA, Rahman A, Dikalov S, Goronzy J, Weyand C, Harrison DG (2007). Role of the T cell in the genesis of angiotensin II–induced hypertension and vascular dysfunction. J Exp Med.

[CR28] Harada M, Kamimura D, Arima Y, Kohsaka H, Nakatsuji Y, Nishida M, Atsumi T, Meng J, Bando H, Singh R (2015). Temporal expression of growth factors triggered by epiregulin regulates inflammation development. J Immunol.

[CR29] Hasebe R, Murakami K, Harada M, Halaka N, Nakagawa H, Kawano F, Ohira Y, Kawamoto T, Yull FE, Blackwell TS, et al. ATP spreads inflammation to other limbs through crosstalk between sensory neurons and interneurons. J Exp Med. 2022;219(6):e20212019. 10.1084/jem.20212019. 10.1084/jem.20212019 PMC9117706 35579694

[CR30] Hirota K, Yoshitomi H, Hashimoto M, Maeda S, Teradaira S, Sugimoto N, Yamaguchi T, Nomura T, Ito H, Nakamura T (2007). Preferential recruitment of CCR6-expressing Th17 cells to inflamed joints via CCL20 in rheumatoid arthritis and its animal model. J Exp Med.

[CR31] Holzer P (1988). Local effector functions of capsaicin-sensitive sensory nerve endings: involvement of tachykinins, calcitonin gene-related peptide and other neuropeptides. Neuroscience.

[CR32] Holzer P (1991). Capsaicin: cellular targets, mechanisms of action, and selectivity for thin sensory neurons. Pharmacol Rev.

[CR33] Huseby ES, Liggitt D, Brabb T, Schnabel B, Ohlén C, Goverman J (2001). A pathogenic role for myelin-specific CD8(+) T cells in a model for multiple sclerosis. J Exp Med.

[CR34] Jager A, Dardalhon V, Sobel RA, Bettelli E, Kuchroo VK (2009). Th1, Th17, and Th9 effector cells induce experimental autoimmune encephalomyelitis with different pathological phenotypes. J Immunol.

[CR35] Kamimura D, Tanaka Y, Hasebe R, Murakami M (2020). Bidirectional communication between neural and immune systems. Int Immunol.

[CR36] Kaufmann WE, Moser HW. Dendritic anomalies in disorders associated with mental retardation. Cerebral Cortex (New York, NY : 1991). 2000;10(10):981–91. Available at 10.1093/cercor/10.10.981. 10.1093/cercor/10.10.981 11007549

[CR37] Kenney MJ, Ganta CK (2014). Autonomic nervous system and immune system interactions. Compr Physiol.

[CR38] Kidd BL, Mapp PI, Gibson SJ, Polak JM, O'Higgins F, Buckland-Wright JC, Blake DR (1989). A neurogenic mechanism for symmetrical arthritis. Lancet (London, England).

[CR39] Kim HR, Cho ML, Kim KW, Juhn JY, Hwang SY, Yoon CH, Park SH, Lee SH, Kim HY (2007). Up-regulation of IL-23p19 expression in rheumatoid arthritis synovial fibroblasts by IL-17 through PI3-kinase-, NF-kappaB- and p38 MAPK-dependent signalling pathways. Rheumatology (Oxford, England).

[CR40] Kivisakk P, Mahad DJ, Callahan MK, Trebst C, Tucky B, Wei T, Wu L, Baekkevold ES, Lassmann H, Staugaitis SM (2003). Human cerebrospinal fluid central memory CD4+ T cells: evidence for trafficking through choroid plexus and meninges via P-selectin. Proc Natl Acad Sci U S A.

[CR41] Korn T, Kallies A (2017). T cell responses in the central nervous system. Nat Rev Immunol.

[CR42] Langrish CL, Chen Y, Blumenschein WM, Mattson J, Basham B, Sedgwick JD, McClanahan T, Kastelein RA, Cua DJ (2005). IL-23 drives a pathogenic T cell population that induces autoimmune inflammation. J Exp Med.

[CR43] Le Borgne M, Etchart N, Goubier A, Lira SA, Sirard JC, van Rooijen N, Caux C, Ait-Yahia S, Vicari A, Kaiserlian D (2006). Dendritic cells rapidly recruited into epithelial tissues via CCR6/CCL20 are responsible for CD8+ T cell crosspriming in vivo. Immunity.

[CR44] Lee J, Nakagiri T, Oto T, Harada M, Morii E, Shintani Y, Inoue M, Iwakura Y, Miyoshi S, Okumura M (2012). IL-6 amplifier, NF-kappaB-triggered positive feedback for IL-6 signaling, in grafts is involved in allogeneic rejection responses. J Immunol.

[CR45] Liston A, Kohler RE, Townley S, Haylock-Jacobs S, Comerford I, Caon AC, Webster J, Harrison JM, Swann J, Clark-Lewis I (2009). Inhibition of CCR6 function reduces the severity of experimental autoimmune encephalomyelitis via effects on the priming phase of the immune response. J Immunol.

[CR46] Locatelli G, Wortge S, Buch T, Ingold B, Frommer F, Sobottka B, Kruger M, Karram K, Buhlmann C, Bechmann I (2012). Primary oligodendrocyte death does not elicit anti-CNS immunity. Nat Neurosci.

[CR47] Louveau A, Harris TH, Kipnis J (2015). Revisiting the Mechanisms of CNS Immune Privilege. Trends Immunol.

[CR48] Lull ME, Block ML (2010). Microglial activation and chronic neurodegeneration. Neurotherapeutics.

[CR49] Mai JK, Paxinos G. The human nervous system, 3rd Ed.: Academic Press; 2011.

[CR50] McKlveen JM, Myers B, Herman JP (2015). The medial prefrontal cortex: coordinator of autonomic, neuroendocrine and behavioural responses to stress. J Neuroendocrinol.

[CR51] Miossec P, Kolls JK (2012). Targeting IL-17 and TH17 cells in chronic inflammation. Nat Rev Drug Discov.

[CR52] Miyazaki K, Itoh N, Ohyama S, Kadota K, Oishi K (2013). Continuous exposure to a novel stressor based on water aversion induces abnormal circadian locomotor rhythms and sleep-wake cycles in mice. PloS One.

[CR53] Morey JN, Boggero IA, Scott AB, Segerstrom SC (2015). Current Directions in Stress and Human Immune Function. Curr Opin Psychol.

[CR54] Murakami M, Okuyama Y, Ogura H, Asano S, Arima Y, Tsuruoka M, Harada M, Kanamoto M, Sawa Y, Iwakura Y (2011). Local microbleeding facilitates IL-6- and IL-17-dependent arthritis in the absence of tissue antigen recognition by activated T cells. J Exp Med.

[CR55] Ogura H, Murakami M, Okuyama Y, Tsuruoka M, Kitabayashi C, Kanamoto M, Nishihara M, Iwakura Y, Hirano T (2008). Interleukin-17 promotes autoimmunity by triggering a positive-feedback loop via interleukin-6 induction. Immunity.

[CR56] Organisciak DT, Vaughan DK (2010). Retinal light damage: mechanisms and protection. Prog Retin Eye Res.

[CR57] Raja SN, Carr DB, Cohen M, Finnerup NB, Flor H, Gibson S, Keefe FJ, Mogil JS, Ringkamp M, Sluka KA (2020). The revised International Association for the Study of Pain definition of pain: concepts, challenges, and compromises. Pain.

[CR58] Ransohoff RM, Engelhardt B (2012). The anatomical and cellular basis of immune surveillance in the central nervous system. Nat Rev Immunol.

[CR59] Ren K, Dubner R (2010). Interactions between the immune and nervous systems in pain. Nat Med.

[CR60] Roth BL (2016). DREADDs for Neuroscientists. Neuron.

[CR61] Sakaguchi N, Takahashi T, Hata H, Nomura T, Tagami T, Yamazaki S, Sakihama T, Matsutani T, Negishi I, Nakatsuru S (2003). Altered thymic T-cell selection due to a mutation of the ZAP-70 gene causes autoimmune arthritis in mice. Nature.

[CR62] Schwartz N, Stock AD, Putterman C (2019). Neuropsychiatric lupus: new mechanistic insights and future treatment directions. Nat Rev Rheumatol.

[CR63] Shen F, Hu Z, Goswami J, Gaffen SL (2006). Identification of common transcriptional regulatory elements in interleukin-17 target genes. J Biol Chem.

[CR64] Steinman L (2014). Immunology of relapse and remission in multiple sclerosis. Annu Rev Immunol.

[CR65] Stofkova A, Kamimura D, Ohki T, Ota M, Arima Y, Murakami M (2019). Photopic light-mediated down-regulation of local alpha(1A)-adrenergic signaling protects blood-retina barrier in experimental autoimmune uveoretinitis. Sci Rep.

[CR66] Stromnes IM, Goverman JM (2006). Passive induction of experimental allergic encephalomyelitis. Nat Protoc.

[CR67] Thakur ER, Sansgiry S, Kramer JR, Waljee AK, Gaidos JK, Feagins LA, Govani SM, Dindo L, El-Serag HB, Hou JK (2020). The Incidence and Prevalence of Anxiety, Depression, and Post-traumatic Stress Disorder in a National Cohort of US Veterans With Inflammatory Bowel Disease. Inflamm Bowel Dis.

[CR68] Theofilopoulos AN, Dixon FJ (1985). Murine models of systemic lupus erythematosus. Adv Immunol.

[CR69] Theoharides TC, Singh LK, Boucher W, Pang X, Letourneau R, Webster E, Chrousos G (1998). Corticotropin-releasing hormone induces skin mast cell degranulation and increased vascular permeability, a possible explanation for its proinflammatory effects. Endocrinology.

[CR70] Tsokos GC (2011). Systemic lupus erythematosus. N Engl J Med.

[CR71] Tuncel J, Haag S, Hoffmann MH, Yau AC, Hultqvist M, Olofsson P, Backlund J, Nandakumar KS, Weidner D, Fischer A (2016). Animal Models of Rheumatoid Arthritis (I): Pristane-Induced Arthritis in the Rat. PloS One.

[CR72] Uchida M, Yamamoto R, Matsuyama S, Murakami K, Hasebe R, Hojyo S, Tanaka Y, Murakami M (2022). Gateway reflexes, neuronal circuits that regulate the autoreactive T cells in organs having blood barriers. Int Immunol.

[CR73] Ulrich-Lai YM, Herman JP (2009). Neural regulation of endocrine and autonomic stress responses. Nat Rev Neurosci.

[CR74] Vogelweid CM, Johnson GC, Besch-Williford CL, Basler J, Walker SE (1991). Inflammatory central nervous system disease in lupus-prone MRL/lpr mice: comparative histologic and immunohistochemical findings. J Neuroimmunol.

[CR75] Willemze RA, Welting O, van Hamersveld HP, Meijer SL, Folgering JHA, Darwinkel H, Witherington J, Sridhar A, Vervoordeldonk MJ, Seppen J, et al. Neuronal control of experimental colitis occurs via sympathetic intestinal innervation. Neurogastroenterol Motil. 2018;30(3):e13163. 10.1111/nmo.13163. 10.1111/nmo.13163 28745812

[CR76] Yednock TA, Cannon C, Fritz LC, Sanchez-Madrid F, Steinman L, Karin N (1992). Prevention of experimental autoimmune encephalomyelitis by antibodies against alpha 4 beta 1 integrin. Nature.

